# Sex Determination across Evolution: Connecting the Dots

**DOI:** 10.1371/journal.pbio.0030021

**Published:** 2005-01-18

**Authors:** Eric S Haag, Alana V Doty

## Abstract

Sexual differentiation appears to be an ancient, and potentially homologous, feature of animal biology, and yet the pathways that underlie the process exhibit bewildering variety

Evolutionary developmental biology is motivated by the premise that the differences we see between species are caused by changes that have occurred in the genes that regulate their developmental programs. Beginning in the 1980s, general principles began to emerge about the evolution of development in animals. The identification of the Hox genes in Drosophila melanogaster and the subsequent discovery of their conservation and similar expression in different Metazoans led to the revolutionary realization that many of the mechanisms critical to basic animal development have been conserved across more than 500 million years of evolution. Many other developmental pathways, such as those specifying the heart and the central nervous system, have since been elucidated and promptly subjected to successful comparative analysis.

These celebrated discoveries illustrate ways that very different organisms are, at a fundamental level, similar to one another. But not all developmental processes are so conservative; an outstanding example is sex determination. The majority of animal species produce two sexes, and current phylogenies (e.g., [1]) suggest that sexual dimorphism was likely a feature of the last common ancestor of the coelomate bilaterians, a vast clade of animals that excludes only sponges, ctenophores, cnidarians, and acoel flatworms. However, though critical for development and reproduction, the mechanisms that specify sex determination are among the least-conserved known. Marked variation exists in both the primary sex determination signal and in the downstream genetic pathways that interpret the signal. We are thus presented with our first conundrum: sexual differentiation appears to be an ancient, and potentially homologous, feature of animal biology, yet its genetic specification suggests multiple origins.

## Bewildering Variety

The variety of primary sex determination cues was appreciated long before the advent of molecular genetics [2]. The two broadest categories are genetic sex determination (GSD), in which the sex of offspring is set by a sex chromosome or an autosomal gene, and environmental sex determination (ESD), in which sex is determined by temperature (as with turtles), local sex ratio (as with some tropical fish), or population density (as with mermithid nematodes). Though little is known about the molecular mechanisms of ESD, within the GSD systems many different mechanisms have been uncovered. Dual sex chromosome systems, in which either the female (ZW/ZZ) or the male (XX/XY) is heterogametic, are common, as are systems set by the ratio of the number of X chromosomes to sets of autosomes (X:A). There are also systems in which heterozygosity at a single locus is required for female development (known as complementary sex determination; [3]), as well as systems involving sex determination via multiple genes with additive effects.

Molecular genetic investigations of GSD in model systems such as Drosophila, Caenorhabditis, and mice have revealed a clear lack of conservation, underscoring the diversity. For example, although the primary sex determination signal in both D. melanogaster and C. elegans is the X:A ratio, the fruit fly pathway consists of a cell-autonomous cascade of regulated mRNA splicing, while that of the nematode follows a Hedgehog-like intercellular signaling pathway [4]. GSD in mammals depends (with some interesting exceptions—see [5]) upon a Y-specific dominant gene (Sry) encoding a transcription factor. In the face of such impressive differences, perhaps we should question our assumption of homology: could it be that sex determination in different taxa has arisen independently over and over again in evolution? Until 1998, this seemed like a good bet.

**Figure 1:**
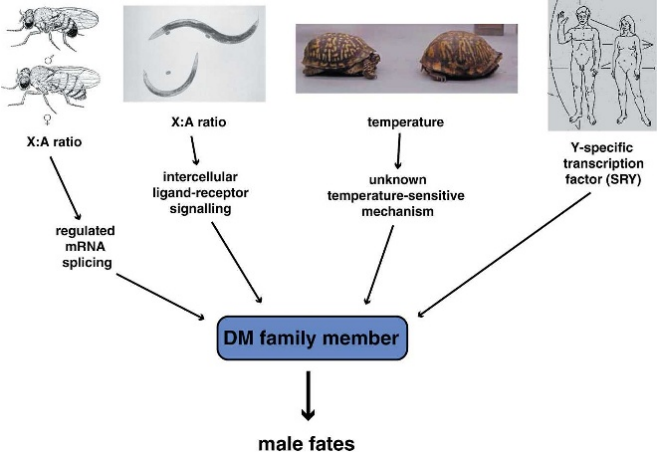
Diverse Genetic Factors Converge on a Conserved Regulator The primary sex determination mechanisms are shown, from left to right, for Drosophila, Caenorhabditis, the box turtle Terrapene carolina, and humans. These proximate signals are then relayed by diverse signal transduction pathways that ultimately converge on a DM-family gene. The left image is from Muller [23]; the center-left image appears courtesy of Dr. Barbara Conradt, the center-right image appears courtesy of J.D. Willson, and the right image is from a plaque mounted on the NASA spacecraft Pioneer 11.

The discovery of the homology of the key sex-determining genes doublesex in Drosophila and *mab-3* in C. elegans provided the first evidence for a common evolutionary basis of sex determination in animals [6]. Soon, related *doublesex-mab-3* (DM)-family genes with roles in male sexual development were discovered in vertebrates and even cnidarians [7,8]. Here at last was a smoking gun that could link the diverse metazoan sex determination systems (Figure 1). But as satisfying as the result was, it immediately gave birth to another mystery: if the enormous diversity of sex determination systems are all derived from a common ancestor, how could they possibly have been modified so radically? After all, sexual differentiation and reproduction are hardly unimportant developmental processes!

## Focusing on Close Relatives

To understand how such diversity came to be, we need to look at the differences between closely related species. This approach allows the discovery and interpretation of small-scale sex determination changes before they are obscured by subsequent changes. The processes discovered in this way might then be reasonably extrapolated to explain the seemingly unrelated systems of more deeply diverged taxa. Work in dipterans [9] and nematodes [10] has revealed three evolutionary phenomena that characterize shorter-term sex determination evolution.

**Figure 2:**
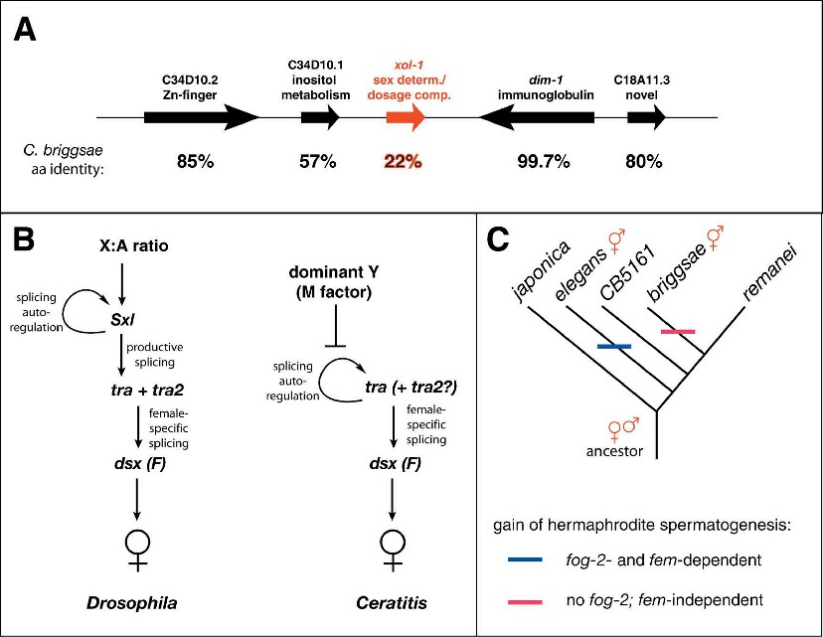
Evolutionary Dynamics of Sex-Determination Pathways (A) Rapid sequence evolution. Shown are the genes in *xol-1* region of C. elegans that have syntenic homologues in C. briggsae, with the amino-acid-level identity between them indicated below. (B) Pathway evolution and primary signal swapping (modified from Graham et al. [9]). In Drosophila (L), the X:A ratio indirectly regulates *tra* splicing through a requirement for *Sxl*. In the medfly Ceratitis (R), *Sxl* is not a sex determination gene, and the female-promoting positive regulation of *tra* is instead autonomous. Its inhibition by the dominant M gene allows an XX/XY system to replace one based on the X:A ratio. (C) Convergent evolution of nematode hermaphroditism in C. elegans and C. briggsae. *fog-2* exists only in C. elegans, and although all species use the *fem* genes for male somatic development, only C. elegans requires them for hermaphrodite spermatogenesis.

The first of these is the often astounding rate of molecular evolution at the level of nucleotide and aminoacid sequences. Although some sex-determining genes are well conserved, many show unprecedented substitution rates [11]. An extreme example is the central integrator of the X:A ratio in Caenorhabditis, *xol-1*. The *xol-1* orthologues of the closely related nematodes C. elegans and C. briggsae are a mere 22% identical [12], even though genes surrounding *xol-1* are much better conserved (Figure 2A). Remarkably, the 3′ neighbor of *xol-1*, the immunoglobulin *dim-1*, is only 5 kb away and is essentially identical between species.

A second phenomenon, best exemplified by dipteran insects, is the modification of genetic control pathways through the gain or loss of key pathway components (Figure 2B). In Drosophila, the first gene to respond to the X:A ratio is *Sxl*, whose transcription is regulated by both autosomal and X-linked factors very early in development [4,13]. When X: A = 1 (i.e., in female embryos), *Sxl* transcription occurs and produces Sxl protein. Later in development, transcription from a second promoter occurs in both sexes, but these transcripts cannot be productively spliced without the earlier burst of *Sxl* expression. As a result, only females sustain *Sxl* expression, and in turn only females can productively splice the mRNA of *tra*, its downstream target. Productive splicing of *tra* is required to produce the female-specific form of *dsx*, a founding member of the DM family mentioned above.

In a series of groundbreaking papers, Saccone and colleagues investigated the pathway in the more distantly related heterogametic Mediterranean fruit fly Ceratitis capitata. The first surprise was that although a highly conserved *Sxl* homologue exists in Ceratitis, it does not undergo sex-specific regulation similar to that of Drosophila, which suggests that it does not play a key switch role (Saccone et al. 1998). Similar results have also been found for the housefly, Musca domestica [14], indicating that the role of *Sxl* in sex determination may be restricted to Drosophila and its closest relatives. In contrast, *tra* and *dsx* are key sex regulators in all dipterans examined thus far.

A further surprise came when the Ceratitis tra homologue was characterized [15]. In the case of this gene, clear evidence for sex-specific regulation was found, and as with Drosophila, only females productively splice *tra* mRNA. However, this splicing difference can be explained nicely by a positive feedback, similar to that seen in Drosophila Sxl, in which Tra protein regulates its own splicing. In 2002, Pane et al. proposed that the dominant, male-specifying M factor on the Y chromosome inhibits this autoregulation [15]. As a result, males cannot make functional Tra protein, and the male form of Dsx is produced. These experiments show not only how a pathway can evolve, but also, importantly, how X:A and heterogametic GSD systems can be interconverted by modifying the cue that regulates a conserved molecular switch gene (the splicing of *tra* mRNA). A detailed scenario for how this might occur has recently been proposed [16].

Finally, recent studies of Caenorhabditis nematodes have shed light on the genetic basis of the convergent evolution of sex determination related to mating system adaptations. An important factor in this area are new phylogenies of the genus [17,18], which consistently suggest the surprising possibility that the closely related hermaphroditic species C. elegans and C. briggsae acquired self-fertilization independently, from distinct gonochoristic (male/female) ancestors (Figure 2C). Although this scenario is somewhat uncertain purely on parsimony grounds, recent work on the genetic control of the germline bisexuality that defines hermaphroditism has tipped the balance toward parallel evolution.

Working with C. elegans, Clifford et al. [19] cloned *fog-2*, a gene required for spermatogenesis in hermaphrodites but not in males. Upon doing so, it became clear that *fog-2* is part of a large family of F-box genes and was produced by several recent rounds of gene duplication. The C. briggsae genome sequence suggested that while C. briggsae possesses a similarly large family of F-box proteins, the duplication event giving rise to *fog-2* was specific to the C. elegans lineage. In this issue of *PLoS Biology*, Nayak et al. [20] extend this work by rigorously demonstrating that *fog-2* is indeed absent in C. briggsae. The authors also identify a short, C-terminal domain that makes FOG-2 uniquely able to perform its germline sex-determining function. This domain is probably derived from a frame-shifting mutation in an ancestral gene. Working with C. briggsae, Stothard et al. [21], Haag et al. [22], and Hill et al. (unpublished data) have also found evidence of important species-specific regulation of germline sex determination. RNA interference and gene knockout approaches have shown that while C. elegans requires the male-promoting genes *fem-2* and *fem-3* to produce sperm in hermaphrodites, C. briggsae requires neither. Given that both genes have conserved roles in male somatic sex determination, this suggests that C. briggsae evolved hermaphroditism in a way that bypasses these genes.

The long-standing mystery of sex determination and its diversity began by comparisons between distantly related species. Recent work on closer relatives has uncovered processes that through a reasonable extrapolation enable the connection of these disparate dots into a fascinating picture of developmental evolution. Though the divergence is extreme, it is likely that a better understanding of the evolution of sex determination genes and pathways holds lessons about the evolution of development in general. The next major challenge will be to integrate the comparative developmental data with the ecological and population processes that are driving the evolution of sex determination. Only then will we be able to say that the picture is complete.

## Abbreviations

DM: *doublesex-MAB-3*
ESD: environmental sex determination
GSD: genetic sex determination
